# LRSAM1 Depletion Affects Neuroblastoma SH-SY5Y Cell Growth and Morphology: The *LRSAM1* c.2047-1G>A Loss-of-Function Variant Fails to Rescue The Phenotype

**DOI:** 10.22074/cellj.2018.5352

**Published:** 2018-05-28

**Authors:** Anna Minaidou, Paschalis Nicolaou, Kyproula Christodoulou

**Affiliations:** 1 Department of Neurogenetics, The Cyprus Institute of Neurology and Genetics, Nicosia, Cyprus; 2 Cyprus School of Molecular Medicine, Nicosia, Cyprus

**Keywords:** Cell Growth, LRSAM1, RING Domain

## Abstract

**Objective:**

Deleterious variants in LRSAM1, a RING finger ubiquitin ligase which is also known as TSG101-associated ligase (TAL), have recently been associated with Charcot-Marie-Tooth disease type 2P (CMT2P). The mechanism by which mutant LRSAM1 contributes to the development of neuropathy is currently unclear. The aim of this study was to induce LRSAM1 deficiency in a neuronal cell model, observe its effect on cell growth and morphology and attempt to rescue the phenotype with ancestral and mutant *LRSAM1* transfections.

**Materials and Methods:**

In this experimental study, we investigated the effect of *LRSAM1* downregulation on neuroblastoma SH-SY5Y cells by siRNA technology where cells were transfected with siRNA against LRSAM1. The effects on the expression levels of TSG101, the only currently known LRSAM1 interacting molecule, were also examined. An equal dosage of ancestral or mutant *LRSAM1* construct was transfected in LRSAM1-downregulated cells to investigate its effect on the phenotype of the cells and whether cell proliferation and morphology could be rescued.

**Results:**

A significant reduction in TSG101 levels was observed with the downregulation of *LRSAM1*. In addition, *LRSAM1* knockdown significantly decreased the growth rate of SH-SY5Y cells which is caused by a decrease in cell proliferation. An effect on cell morphology was also observed. Furthermore, we overexpressed the ancestral and the c.2047-1G>A mutant *LRSAM1* in knocked down cells. Ancestral *LRSAM1* recovered cell proliferation and partly the morphology, however, the c.2047-1G>A mutant did not recover cell proliferation and further aggravated the observed changes in cell morphology.

**Conclusion:**

Our findings suggest that depletion of *LRSAM1* affects neuroblastoma cells growth and morphology and that overexpression of the c.2047-1G>A mutant form, unlike the ancestral *LRSAM1*, fails to rescue the phenotype.

## Introduction

Leucine Rich Repeat And Sterile Alpha Motif Containing 1 (Fig LRSAM1), a RING finger E3 ubiquitin ligase, participates in a range of cellular functions including cell adhesion, signaling pathways and cargo sorting through receptor endocytosis (1, 2), and is expressed in both fetal and adult nervous systems (3). LRSAM1, also known as TSG101-associated ligase (TAL), regulates the metabolism of Tumor Susceptibility gene 101 (*TSG101*) by attaching several monomeric ubiquitins to the C-terminus of TSG101 (4). *TSG101* is a tumor suppressor gene and a component of the endosomal sorting complex required for transport (ESCRT) machinery, with a significant role in cell cycle regulation and differentiation, and is the only reported interactor of LRSAM1 (2, 5).

Ubiquitination is an important process of the cellular system, primarily regulating protein degradation by proteasomes, endocytosis, transcription regulation, protein trafficking and cell death (6, 7). Necessary components of ubiquitination are three highly specific enzymes, namely ubiquitin-activating enzyme (E1), ubiquitin-conjugating enzyme (E2) and ubiquitin protein ligase (E3) (8). E2 enzymes form a complex with ubiquitin (E2-Ub) that is pre-activated by E1 enzymes. The formed E2-Ub complex is recognized and coupled by E3 ligases for ubiquitin transfer to the target site (9). It has become evident that E3 ubiquitin ligases play an essential role in the regulation of axons, dendrites and dendritic spine morphogenesis as well as in neuronal function (7, 10). Aberrant E3 ubiquitin ligase activity has been associated with neurological disorders, including mutations in *PARK2* (Parkinson Protein 2) and *LRSAM1* which result in the juvenile type of Parkinson’s disease (10) and the Charcot-Marie-Tooth disease respectively (4). 

*LRSAM1* has recently been implicated in Charcot- Marie-Tooth (CMT) pathways but its role remains unclear. *LRSAM1* knockdown in zebrafish has been reported to cause delayed neurodevelopment (3), while *LRSAM1* knockout mice presented only sensitization to acrylamideinduced neurodegeneration with no anatomical or functional abnormalities (11). Transfection of the c.2080C>T *LRSAM1* variant in *LRSAM1* knockout NSC34 mouse neuronal cells caused axonal degeneration and also disrupted interaction of LRSAM1 with RNA-binding proteins (12). 

We reported a dominant *LRSAM1* mutation (c.20471G> A, p.Ala683ProfsX3) in a large Sardinian CMT type 2P family (13). To date, five other *LRSAM1* mutations have been associated with CMT neuropathy of which one and four show recessive (4) and dominant (3, 12, 14, 15) inheritance respectively. All the dominant mutations identified are located within the RING finger domain whereas the recessive mutation is located 37 amino acids upstream of this domain. To functionally examine the effect of the dominant variant we had identified, we downregulated *LRSAM1* in neuroblastoma SH-SY5Y cells and overexpressed the ancestral and the c.20471G> A mutant *LRSAM1* in *LRSAM1* knocked down cells. We hereby report the effects of *LRSAM1* downregulation on SH-SY5Y cells and the inability of mutant *LRSAM1* to reverse the phenotype, contrary to the ancestral form. 

## Materials and Methods

In this experimental study, we investigated the effect of *LRSAM1* downregulation on neuroblastoma SH-SY5Y cells followed by the overexpression of ancestral and mutant LRSAM1 in these knocked down cells. 

### Human SH-SY5Y neuroblastoma cells culture

Human SH-SY5Yneuroblastoma cells (ECACC, Sigma- Aldrich, USA) were cultured in DMEM (Invitrogen, USA) without L-glutamine. The growth medium was supplemented with 10% fetal bovine serum (FBS, Invitrogen, USA), 2% GlutaMAX^TM^ (Gibco, USA) and 1% Penicillin-Streptomycin 100X Solution (Invitrogen, USA). SH-SY5Y cell lines were incubated at 37°C under 5% CO_2_. 

### Plasmid construction

#### Whole human LRSAM1 constructs

The pIRES2-EGFP-*LRSAM1* ancestral and mutant constructs were purchased from Eurofins (Germany). Vectors (pIRES2-EGFP) contained the whole coding and part of the 5´ and 3´ UTR of the human *LRSAM1* cDNA in frame. NheI and EcoRI restriction sites were added at the two ends. The mutant *LRSAM1* cDNA included a G base deletion at the first codon of exon 25, representing the frameshift effect of the c.2047-1G>A splice defect variant (13). 

### Transfection

Cells were transfected using Lipofectamine® 3000 (Life Technologies, USA) according to the manufacturer’s instructions. Lipofectamine® 3000 along with either constructs or siRNA were dissolved in the Opti-MEM® (Life Technologies, USA) reduced serum medium without FBS and antibiotics. 

### Downregulation of *LRSAM1* in neuroblastoma SH-SY5Y cells

For downregulation of *LRSAM1*, cells were doubletransfected with siRNA (Table 1) to ensure a more efficient reduction of gene expression. The siRNA against *LRSAM1* (Life Technologies, USA) was transfected into SH-SY5Y cells according to the manufacturer’s instructions. Negative control siRNA (Life Technologies, USA), lipofectamine only and untransfected-A were used as controls of the experiments. Untransfected-A represented cells that had not been transfected with siRNA while the other two controls were used to detect any effect of transfection. The cell growth rate was observed every 24 hours (Table 1). Protein extraction was undertaken on days 2 and 5.

### Transfection of *LRSAM1* constructs in knocked down SH-SY5Y cells 

Knocked down SH-SY5Y cells were transfected on day 5 (Table 1), using an equal, maximum recommended, dose of ancestral or mutant *LRSAM1* construct. The empty vector (pIRES2-EGFP) was used as a control to test for any possible effect of the construct on the cells. Untransfected-B cells were also used as controls which were treated with *LRSAM1* siRNA but not transfected with either ancestral or mutant *LRSAM1* construct. Cells were monitored using the IX73 inverted microscope (Olympus, Japan) before and 48 hours after transfection. 

**Table 1:** *LRSAM1* knockdown timetable


Intervals	Procedure	Counting

Day 0	Platting	√
Day 1-40% cells confluency	1^s^^t^ transfection	√
Day 2-24 hours after 1^s^^t^ transfection	2^n^^d^ transfection	√
Day 3-48 hours after 1^s^^t^-24 hours after 2^n^^d^ transfection	Incubation	√
Day 4-72 hours after 1^s^^t^-48 hours after 2^n^^d^ transfection	Incubation	√
Day 5-96 hours after 1^s^^t^-72 hours after 2^n^^d^ transfection	Incubation	√


### Cell concentration in knocked down SH-SY5Y cells 

Cells were collected from each well using the appropriate volume of 0.25% Trypsin-EDTA (Life Technologies, USA). Concentration of live cells (number of cells/ml) was determined using a hemocytometer (Hausser Scientific, USA). Trypan blue 0.4% (Sigma-Aldrich, USA) was added to the cell suspension (1:1 ratio) and only circular cells that did not absorb the blue dye were counted.

### Protein extraction from SH-SY5Y cells

Proteins were extracted using a protein lysis buffer containing 1M NaCl, 10 mM Tris-Cl (pH=7.5), 10% glycerol (Sigma-Aldrich, USA), 1% Tween^TM^20(Affymetrix, USA), 10 Mm ß-mercaptoethanol (Sigma-Aldrich, USA) and 1x EDTA-free Protease Inhibitor Cocktail (Promega, USA). Lysates were sonicated, denatured at 95°C and diluted in 1x sodium dodecyl sulfate 20 % (Fisher Scientific, UK). The Coomassie Plus (Bradford) protein assay (Thermo Scientific, USA) was used to measure the protein concentration. 

### Western blot analysis

Proteins were separated on SDS-PAGE (8-12%) gels and transferred to PVDF membranes (Millipore, Germany). Membranes were blocked with 5% non-fat milk in PBS0.1% TweenTM20 and were incubated overnight at 4°C with the respective specific primary antibody for each protein [mouse anti-LRSAM1/Abcam ab73113 (1:400), rabbit anti-LRSAM1/Novus Biological H00090678-D01 (1:750), rabbit anti-CASPASE-3/Santa Cruz SC7148 (1:700), mouse anti-CYCLIN D1/Abcam ab6152 (1:300), mouse anti-TSG101/Novus Biological NB200-11 (1:500) and mouse anti-ß-ACTIN/Sigma-Aldrich A2228 (1:4000)] which was diluted in phosphate buffered saline (PBS)-0.1% TweenTM20. The membranes were then incubated for 2 hours with the appropriate secondary antibodies [AP124P goat anti-mouse IgG-Peroxidase H+L/Millipore (1:7000) and sc-2077 donkey anti-rabbit IgG-HRP/ Santa Cruz (1:7000)] followed by incubation with the visualization LumiSensorTM Chemiluminescent HRP Substrate Kit (Genscript, USA). Membranes were finally visualized using the UVP imaging system (BioRad, USA). Western blots were quantified using the ImageJ software (https://imagej.nih.gov). Protein quantity ratio was estimated relative to ß-actin. 

### Statistical analysis 

Quantitative data (ratio %) from three independent experiments were analyzed using the two-tailed Student’s paired t test. A P<0.05 was considered statistically significant. All the data are expressed as mean ± SD of the three replicates. The mean of quantitative data of the control samples was set to 100%.

### Ethical considerations

This study was ethically approved by the Cyprus National Bioethics Committee (ΕΕΒΚ/ΕΠ/2013/28).

## Results

### Downregulation of LRSAM1 affects growth and morphology of undifferentiated SH-SY5Y cells 

**Fig.1:**
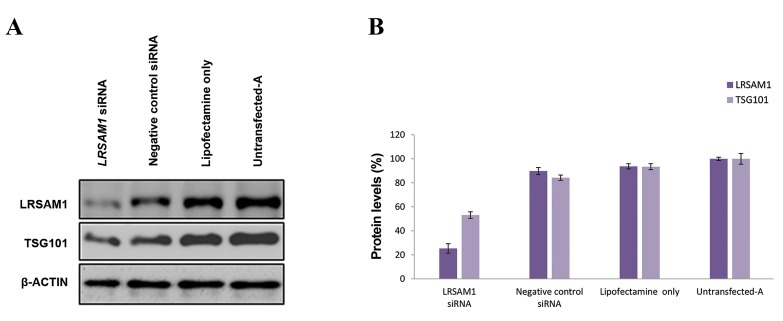
siRNA-mediated downregulation of LRSAM1. A. Western blot analysis of endogenous LRSAM1 and TSG101 levels, compared with thecontrols (negative control siRNA, lipofectamine only and untransfected-A cells). LRSAM1 and TSG101 levels were determined 72 hours after thesecond transfection. ß-ACTIN was used as an internal control and B. Quantification of LRSAM1 and TSG101 was performed relative to ß-ACTIN. Untransfected-A cell values (LRSAM1 and TSG101) were set to 100% to reflect the normal cell growth within the 5 days of the experimentalprocedure. LRSAM1 levels (purple colour): LRSAM1 siRNA transfected cells (25 ± 3.94%, P<0.004), negative control siRNA transfected cells (90 ± 2.89%, P<0.009), lipofectamine only transfected cells (94 ± 2.15%, P<0.008) and untransfected-A cells (100 ± 1.36%). TSG101 levels (light purplecolour): *LRSAM1* siRNA transfected cells (53 ± 2.72%, P<0.007), negative control siRNA transfected cells (84 ± 2.20%, P<0.027), lipofectamine onlytransfected cells (93 ± 2.49%, P<0.040) and untransfected-A cells (100 ± 4.51%).

**Fig.2:**
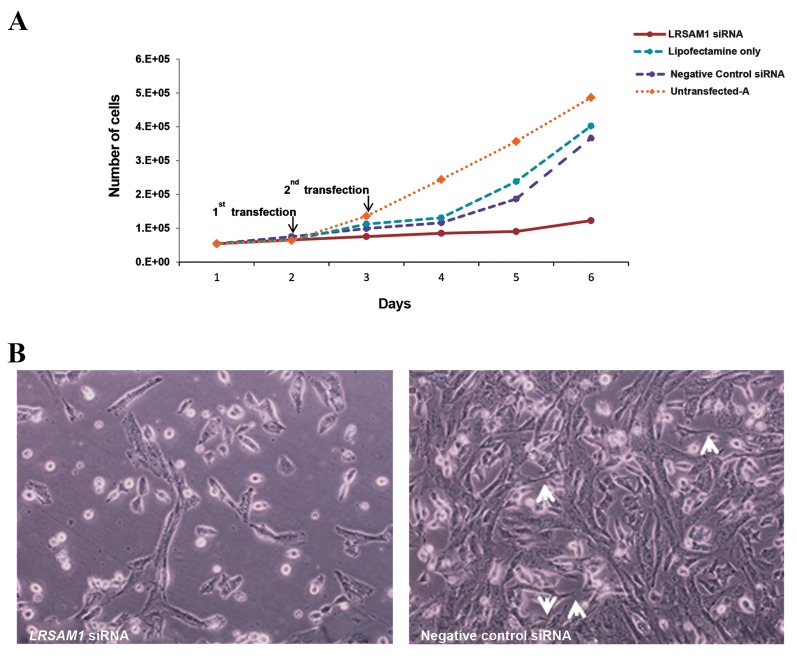
Effect of *LRSAM1* knockdown on SH-SY5Y cells. A. Double transfection of *LRSAM1* siRNA was performed on days one and two of the experimental procedure. Cell counts after every 24 hours from day zero (plating) are depicted. Negative control siRNA, lipofectamine only and untransfected-A cell cultures were performed and used as control for the experiment and B. Microscopy analysis 72 hours after the first LRSAM1 downregulation in double transfected *LRSAM1* siRNA and negative control siRNA SH-SY5Y cells. Arrows heads show the formation of early neurites (scale bar: 200 µm).

The efficiency of downregulation was evaluated with Western blot analysis on days 2 and 5. Twenty-four hours after the first *LRSAM1* siRNA transfection (day 2), we observed a 41 ± 2.94% reduction of endogenous LRSAM1. Downregulation of *LRSAM1* also caused a 30 ± 4.99% reduction in TSG101 levels (data not shown). Following the double-transfection of *LRSAM1* siRNA, a 75 ± 3.94% and 47 ± 2.72% decrease in LRSAM1 and TSG101 levels was observed (Fig .1). Growth of *LRSAM1* knocked down cells was remarkably reduced 72 hours after the second transfection (day 5). Slightly reduced growth was observed in negative siRNA and lipofectamine only controls, reflecting the transfection effect (Fig .2A). However, downregulation of *LRSAM1* substantially decreased the growth rate of neuroblastoma SH-SY5Y cells when compared with the controls (P_LRSAM1/untransfected-A_=0.0478).

Monitoring the cells 72 hours after the first transfection, we observed that *LRSAM1* downregulation had affected the morphology of the cells compared with negative control cells (Fig .2B). The majority of knocked down cells had a spherical shape with only a small proportion displaying an elongated shape. No neurite formation among the cells was observed in knocked down cells. In addition to a higher density, negative control cells not only showed an elongated shape, but they also displayed short neurite outgrowths in empty spaces among the cells with a tendency to form networks.

**Fig.3:**
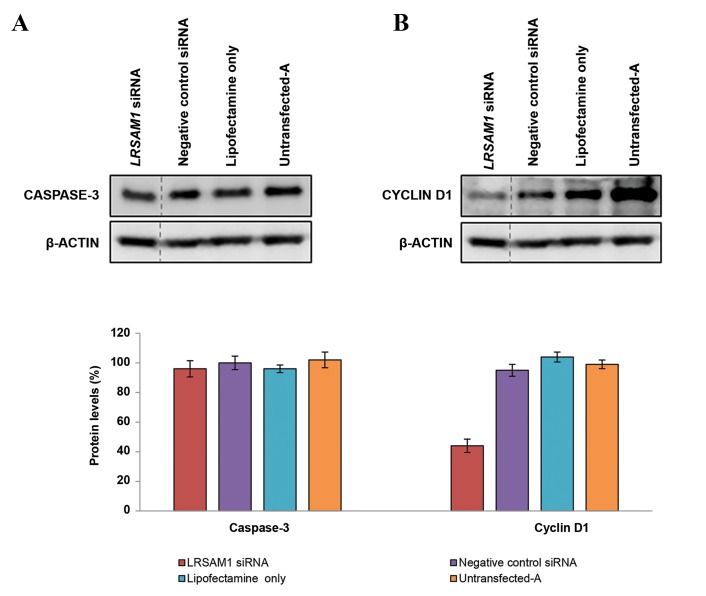
Investigation of apoptotic (caspase-3) and cell cycle (cyclin D1) markers in *LRSAM1* knockdown SH-SY5Y cells. Western blot analysis of A. Caspase-3and B. Cyclin D1 levels compared with the controls (negative control siRNA, lipofectamine only and untransfected-A cells). Protein levels were determined 96 hours after the first transfection. ß-ACTIN was used as an internal control. Quantification of caspase-3 and cyclin D1 was performed relative to ß-ACTIN. Untransfected-A cell values (caspase-3 and cyclin D1) were set to 100%. Caspase-3 levels: *LRSAM1* siRNA transfected cells (96 ± 5.48%, P<0.018), negative control siRNA transfected cells (100 ± 4.55%, P<0.02), lipofectamine only transfected cells (96 ± 2.58%, P<0.019) and untransfected-A cells (102 ± 5.31%). Cyclin D1 levels: *LRSAM1* siRNA transfected cells (44 ± 4.50%, P<0.005), negative control siRNA transfected cells (95 ± 3.99%, P<0.009), lipofectamine only transfected cells (104 ± 3.33%, P<0.03) and untransfected-A cells (99 ± 2.92%).

To identify the cause of the observed reduction in growth rate, we examined the protein levels of two protein markers with Western blot analysis. Cyclin D1 and Caspase-3 were used as the cell cycle and apoptotic markers respectively. Equal expression of Caspase-3 was detected in knocked down and control cells (Fig .3A). However, a significant reduction of 44 ± 4.50% of Cyclin D1 expression was observed in knocked down cells when compared with controls (Fig .3B), thus indicating a potential role for *LRSAM1* in the cell cycle process. 

### Transfection of LRSAM1 in knocked down SH-SY5Y cells

**Fig.4:**
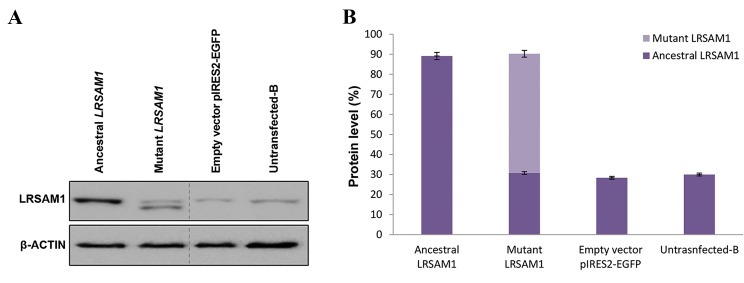
Evaluation of endogenous and exogenous LRSAM1 levels. A. Western blot analysis of *LRSAM1* levels 48 hours after the *LRSAM1* transfection in knocked down SH-SY5Y cells. An approximately 30% of endogenous protein was detected. ß-ACTIN was used as an internal control and B. Quantification of ancestral and mutant LRSAM1 was performed relative to ß-ACTIN. Untransfected-B cell values (LRSAM1) were set to 30% (endogenous LRSAM1 background). Ancestral *LRSAM1* transfected cells representing ancestral-endogenous + ancestral-exogenous LRSAM1 levels within a single band (89 ± 1.80%, P<0.0087 relative to untransfected-B). LRSAM1 mutant transfected cells representing ancestral-endogenous (31 ± 0.68%, P<0.01) and mutant- exogenous (59 ± 1.68%, P<0.007) LRSAM1 levels in two different size bands. Empty vector pIRES2-EGFP transfected cells representing ancestral- endogenous levels (28 ± 0.71%, P<0.0045). Untransfected-B cells representing ancestral-endogenous LRSAM1 levels (30 ± 0.72%, P<0.0019).

Knocked down cells were transfected with ancestral or mutant *LRSAM1* constructs. Efficiency of the transfection was monitored with Western blot analysis (Fig .4). Given that the efficiency of the knockdown was approximately 70%, 30% of the endogenous LRSAM1 protein level was expected to be present. This level of endogenous LRSAM1 was confirmed in the empty vector pIRES2EGFP and the untransfected-B controls as well as those transfected with the mutant *LRSAM1*. In the latter, two bands were observed representing the ancestral (endogenous) and the lower molecular weight truncated mutant protein (exogenous). Total expression levels of LRSAM1 (ancestral and mutant) in ancestral and mutant transfected knocked down cells was equivalent (89 and 90% respectively). 

**Fig.5:**
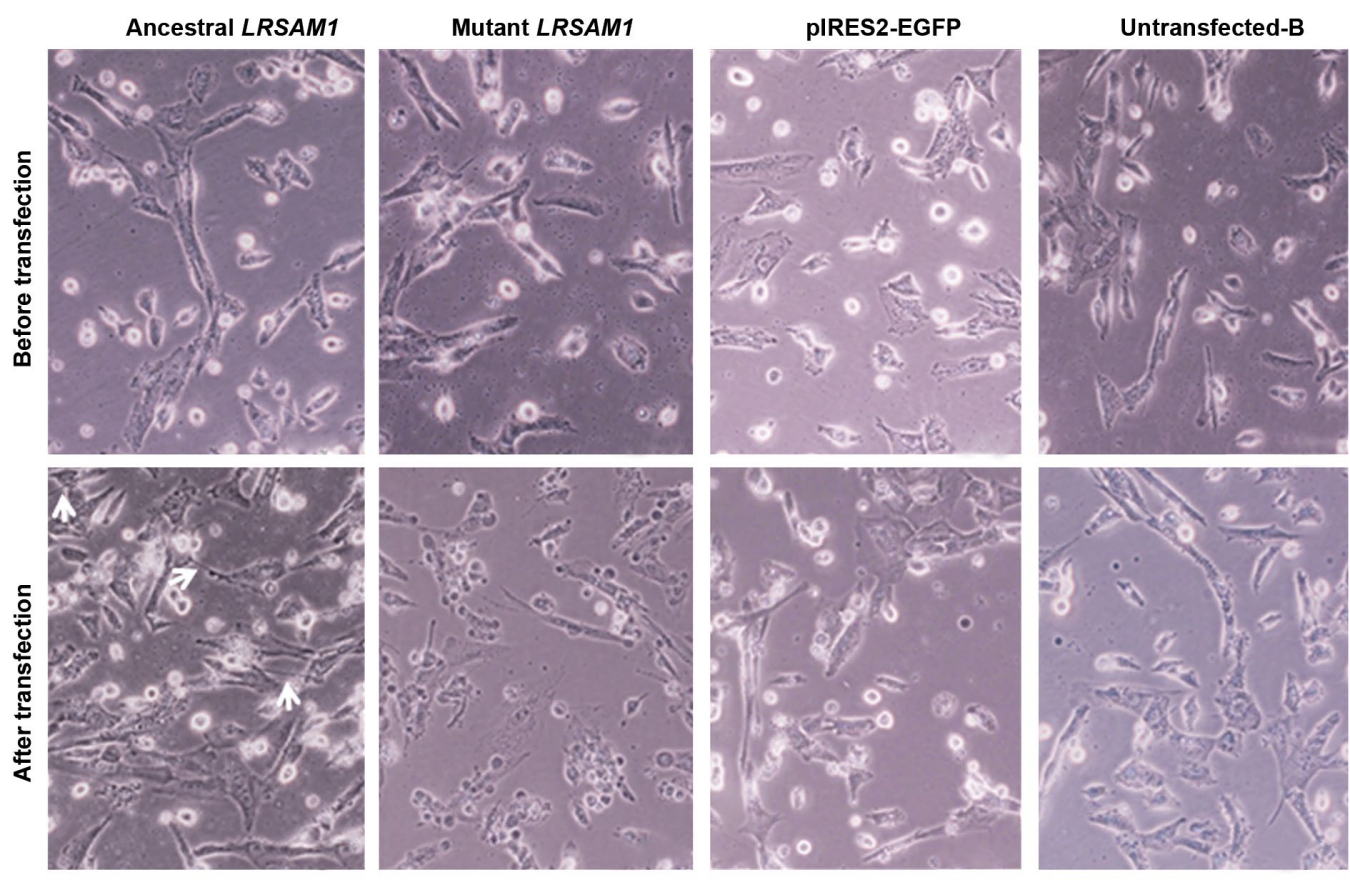
Investigation of cell morphology and proliferation after transfection of ancestral or mutant LRSAM1 in knocked down SH-SY5Y cells. Microscopy analysis before and 48 hours after the transfection of ancestral or mutant LRSAM1 constructs. At the time of LRSAM1 transfection, cells expressed 30% of the endogenous LRSAM1 level. Arrows show the formation of early neurites (scale bar: 100 µm).

We overexpressed ancestral and mutant *LRSAM1* in knocked down cells to investigate whether cell proliferation and morphology could be rescued. Forty- eight hours after transfection of the ancestral *LRSAM1,* the growth of cells was considerably recovered when compared with controls. Cells transfected with ancestral *LRSAM1* re-proliferated, morphology of the cells was partly retrieved and cells displayed a more elongated shape. Also, cells initiated formation of early neurites. Transfection with an equal dose of the mutant *LRSAM1* not only did not improve cell proliferation, but also worsened the morphological features of cells when compared with controls. Specifically, mutant *LRSAM1* transfection induced the formation of cell clusters comprising small circular and thin elongated cells. Empty vector pIRES2-EGFP and untransfected-B cells displayed the same morphology and growth prior to the transfection without any improvement in density, neurite formation and alteration in the shape of the cells (Fig .5). 

## Discussion

A relatively small number of variants in *LRSAM1* have been associated with CMT2P, however, the majority of them are predicted to interfere with the RING domain and in turn the E3 ubiquitin ligase activity of the protein. Ubiquitination plays a central role within cells and its disruption is expected to have an impact on the overall wellbeing of the cell. We thus investigated the effect of LRSAM1 depletion on cell growth and morphology using neuroblastoma cells, a good in vitro model to study the function of a gene that is associated with a neurological condition, namely CMT2P neuropathy. 

LRSAM1 incomplete (70%) knockdown severely affected the growth and morphology of neuroblastoma cells. However, failure to observe a phenotype in mouse NSC34 cells despite complete knockout of *LRSAM1* with the CRISPR/Cas9 system has been reported (11, 12). The mouse model which was homozygous for a loss-of-function variant in *Lrsam1* had no anatomically or functionally detectable abnormalities with only a sensitivity of peripheral motor axons to acrylamideinduced degeneration (11). Unlike the mouse model, the morpholino induced *Lrsam1* zebrafish model had both anatomically and functionally detectable abnormalities (3). Thus, species- and/or cell/model-specific *LRSAM1* regulation may explain the differences between the effects of LRSAM1 depletion that we observed in human neuroblastoma SH-SY5Y cells as opposed to the reported effect in mouse NSC34 cells. 

Given that *LRSAM1* downregulation affected the cell cycle process by observing a lower level of cyclin D1, the G1-phase is most likely impaired. We also observed a significant reduction of TSG101 levels with downregulation of *LRSAM1*. In another study on TSG101 deficient cells, G1 and G1/S-phase cyclins D1 and E were not affected, however the S and M phase cyclins A2 and B1 were prominently decreased (16). We therefore suggest that knockdown of LRSAM1 affects cell cycle regulation in the G1-phase at an earlier stage than TSG101. The two molecules may act directly or indirectly and through interaction with each other or independently to cause changes in the cell cycle. 

LRSAM1, through its E3 ligase activity, regulates the level of TSG101 by targeting it for ubiquitination and degradation. Silencing of *TSG101* has been shown to decrease proliferation and cell growth of adult and embryonic tissues (17). Early embryonic lethality was also observed in homozygous knockout *TSG101* mice (18). Interestingly, strong overexpression of TSG101 also prevented cell division and induced cell cycle arrest (19). It therefore seems that TSG101 is tightly controlled and is necessary for normal cell function with LRSAM1 having a significant role in adjusting the TSG101 level. 

Other E3 ubiquitin ligases have also been reported to be involved in cell cycle regulation and neurodegeneration. Two E3 ubiquitin ligase complexes are involved in cell cycle regulation; SCF (SKP1/CUL1/F-box) and APC/C (anaphase prompting complex) complexes mediate ubiquitination and activation of cell cycle marker proteins (20). In addition, deficiency in the E3 ubiquitin ligase activity of TRIM2 has been reported to cause severe early- onset axonal CMT2R (21). TRIM2 ubiquitinates the neurofilament light chain and patient sural nerve biopsy as well as TRIM2-gene trap mice studies have shown the accumulation of neurofilaments inside the axons (21, 22). Deregulation in the ubiquitin proteasome system (UPS), consisting of E3 ubiquitin ligases, has also been associated with neurodegenerative disorders. Parkin, an E3 ligase, exerts a direct, confirmed impact on neurodegeneration which leads to Parkinson disease (23). Decreased levels of HRD1and Fbxo2 E3 ligases have also been reported in tissues obtained from Alzheimer’s patients (24). 

Other CMT genes, which lack E3 ubiquitin ligase activity, have also been implicated in cell cycle progression. The ancestral *GDAP1* (CMT2K and CMT4A) has been reported to rescue cell cycle delay caused by Fis1 deficiency in contrast to mutant *GDAP1* (25), and an extended cell cycle was observed in zebrafish mutants with impaired *PRPS1* expression, a gene that has been associated with X- linked CMT (26).

In the second part of this study, we overexpressed the ancestral and the c.2047-1G>A mutant *LRSAM1* in knocked down cells. Given that transfection with the mutant form failed to rescue the phenotype and also deteriorated the morphology of the cells, we speculate that the c.2047-1G>A mutation, which leads to protein truncation, dramatically affects the activity of the enzyme rendering it unable to cause ubiquitination. A recent study demonstrated that recombinant mutant LRSAM1 RING proteins, carrying frameshift or missense mutations, abolished the ligase activity of LRSAM1. Mutant proteins failed to interact and form poly-ubiquitin chains with E2 enzymes, which is a crucial process for efficient target ubiquitination (27). Transfection of *LRSAM1* with a missense variant in the RING domain also caused axonal degeneration in LRSAM1 knockout NSC34 cells and disrupted interaction of LRSAM1 with RNA-binding proteins (12). 

Five of the six *LRSAM1* mutations that have been associated with CMT2P exert a dominant effect and are all located within the RING domain, suggesting that the E3 ligase activity of the protein is affected. The recessive mutations are located upstream of the RING domain and result in no detectable protein. A dominant-negative effect of the dominant mutations is possible, with the presence of the mutant protein interfering with the function of the ancestral LRSAM1, given the similar clinical phenotype between patients with dominant and recessive forms. This possibility is supported by the findings of Hakonen et al. (27), who demonstrated that LRSAM1 RING mutants maintained the capacity to form heterodimers with the ancestral LRSAM1 proteins. Another possibility of a dominant-negative effect of mutant LRSAM1 may be through its interaction with TSG101. LRSAM1 interacts with TSG101 for proper ubiquitination of TSG101 (2) and the interacting amino acids precede the RING domain of the protein, and both mutant and ancestral LRSAM1 may therefore compete for TSG101 binding. The interaction and formation of a complex between mutant LRSAM1 and TSG101 is not affected by the disruption of the LRSAM1 RING domain (27). In the absence of ubiquitination efficiency caused by RING domain-mutant LRSAM1, the mutant LRSAM1-TSG101 complex may lead to blockage of the TSG101 ubiquitination pathway, consistent with an alternative dominant-negative effect of mutant LRSAM1. 

## Conclusion

We suggest that depletion of *LRSAM1* affects neuroblastoma cells growth and morphology and that overexpression of the c.2047-1G>A mutant, in contrast to the ancestral *LRSAM1*, fails to rescue the phenotype.
